# MiR-19b-3p facilitates colorectal cancer metastasis by mediating the crosstalk between tumor-associated endothelial cells and malignant cells

**DOI:** 10.3389/fonc.2026.1701701

**Published:** 2026-03-02

**Authors:** Yixin Cao, Jia Zhang, Xinyu Kai, Yongwang Xue, Xuequan Wang, Xiangyang Wang, Cai Zhang, Zhongyi Hua, Yuan Wei, Runting Yin

**Affiliations:** 1 Department of Medical Oncology, Affiliated Hospital of Jiangsu University, Zhenjiang, China; 2 School of Pharmacy, Jiangsu University, Zhenjiang, China; 3 Department of Radiation Oncology, Taizhou Hospital of Zhejiang Province Affiliated to Wenzhou Medical University, Linhai, Zhejiang, China; 4 Department of Traditional Chinese Medicine, Jiangsu University Affiliated People’s Hospital, Zhenjiang Clinical Medical College of Nanjing Medical University, Zhenjiang, China

**Keywords:** colorectal cancer, EMT, EndMT, miR-19b-3p, tumor microenvironment, ZMYND11

## Abstract

**Background:**

The crosstalk between tumor cells and stromal components in the tumor microenvironment critically influences colorectal cancer (CRC) progression and metastasis. The role of tumor-associated endothelial cells (TAECs) in this process is not fully understood. This study aimed to elucidate the function of miR-19b-3p in mediating CRC-endothelial cell communication.

**Methods:**

The effects of miR-19b-3p were investigated in CRC cells (HCT116) and human umbilical vein endothelial cells (HUVECs) using functional assays and molecular analyses. A co-culture model was employed to study intercellular crosstalk. In vivo validation was performed using a mouse peritoneal tumor model. Circulating miR-19b-3p levels were measured in CRC patient samples and correlated with clinicopathological parameters.

**Results:**

miR-19b-3p promoted epithelial-to-mesenchymal transition (EMT) in HCT116 cells by suppressing ZMYND11 and activating the MAPK pathway. In HUVECs, it induced endothelial-to-mesenchymal transition (EndMT) via upregulation of SOX9. Co-culture led to reciprocal elevation of miR-19b-3p in both cell types, indicating a reinforcing bidirectional loop. In mice, miR-19b-3p overexpression increased peritoneal tumor burden and induced EMT markers (loss of E-cadherin, gain of N-cadherin). Clinically, elevated circulating miR-19b-3p in CRC patients was associated with advanced disease stage and poor prognosis.

**Conclusion:**

Our findings reveal that miR-19b-3p orchestrates tumor-endothelial interactions by synchronously promoting EMT and EndMT, thereby driving a pro-metastatic microenvironment. Circulating miR-19b-3p represents a promising biomarker and potential therapeutic target in metastatic colorectal cancer.

## 1 Introduction

Colorectal cancer (CRC) is a malignant tumor originating from intestinal polyps and is characterized by dysregulated signaling pathways that drive oncogenesis and metastasis (1, 2). Globally, CRC accounts for 10.6% of all cancer cases and remains a leading cause of cancer-related death, with a case fatality rate of 9.3%, second only to lung and liver cancers (3). Although the incidence of CRC has declined in elderly populations, recent epidemiological studies reveal an annual increase of approximately 2% among younger individuals (4).

Tumor metastasis is responsible for over 90% of cancer-related deaths (5). A key step in the metastatic cascade involves the detachment of cancer cells from the primary tumor mass, often accompanied by epithelial-to-mesenchymal transition (EMT), a process that confers stem-like properties and enhanced motility (6, 7). In addition to malignant epithelial cells, tumor-associated endothelial cells (TAECs) play an essential role in tumor growth, angiogenesis, and metastatic dissemination (8–10). Within the tumor microenvironment (TME), endothelial cells may undergo phenotypic changes, including endothelial differentiation (11) and endothelial-to-mesenchymal transition (EndMT) (12). While EMT has been reported to promote angiogenesis through vascular endothelial growth factor A (VEGF-A) upregulation (13), whether EMT contributes to the initiation or progression of EndMT remains unclear.

MicroRNAs (miRNAs) are short non-coding RNAs (18–22 nucleotides) that regulate gene expression post-transcriptionally by binding to the 3′ untranslated regions (3′-UTRs) of target mRNAs (14). Numerous studies have implicated miRNAs in tumor progression and metastasis (15, 16), and miRNA-based therapeutic strategies are emerging as promising approaches in oncology (17). MiR-19b-3p, a known oncogenic miRNA transcribed by c-Myc, has been shown to promote EMT in colorectal cancer (18), renal cancer (19), and glioma (20). Interestingly, miR-19b-3p also exhibits endothelial cell-specific activity. Our group previously reported that miR-19b-3p suppresses angiogenesis by targeting fibroblast growth factor receptor 2 (FGFR2) in human endothelial cells (21), and similar anti-angiogenic effects were observed in breast cancer models (22). Furthermore, anti-angiogenesis therapy with bevacizumab has been shown to induce EndMT in breast cancer (23), suggesting a potential link between miR-19b-3p and EndMT regulation.

Although miR-19b-3p has been implicated in CRC progression (18), its role in modulating the tumor vascular microenvironment and the interaction between EMT and EndMT remains poorly understood. Based on these observations, we hypothesized that miR-19b-3p mediates bidirectional crosstalk between colorectal cancer cells and tumor-associated endothelial cells, facilitating both EMT and EndMT processes.

In this study, we demonstrate that miR-19b-3p promotes EMT in CRC cells via direct suppression of the tumor suppressor ZMYND11, leading to activation of the MAPK signaling pathway. Simultaneously, miR-19b-3p induces EndMT in endothelial cells by upregulating SOX9. In a co-culture model of CRC cells and endothelial cells, we observed reciprocal miR-19b-3p elevation, suggesting mutual reinforcement between EMT and EndMT. These findings uncover a novel mechanism of CRC metastasis driven by miR-19b-3p and highlight its potential as a therapeutic target within the tumor microenvironment.

## 2 Materials and methods

Methods such as migration, invasion, and tube formation were adapted from our previously established protocols (22).

### 2.1 Clinical samples

A total of 10 colorectal cancer (CRC) patients and 4 normal controls were enrolled in this study. All participants were recruited from the Affiliated Hospital of Jiangsu University. Written informed consent was obtained from each participant prior to sample collection. The study protocol was reviewed and approved by the Medical Ethics Committee of Jiangsu University (Approval No. KY2021K1004).

Inclusion criteria for CRC patients were as follows:

1. Histologically confirmed colorectal adenocarcinoma.
2. Evidence of peritoneal or distant metastasis confirmed by imaging or intraoperative findings.
3. No prior chemotherapy or radiotherapy before sample collection.
4. Availability of complete clinicopathological and staging data.

Exclusion criteria included:

1. History of other malignancies.
2. Severe systemic comorbidities or autoimmune disease.
3. Incomplete clinical data or poor-quality samples.

The control group consisted of four healthy adult volunteers undergoing routine physical examination, with no history of malignancy, gastrointestinal disorders, or chronic illness.

Peripheral blood samples were collected preoperatively from all CRC patients and healthy controls. In selected CRC patients, tumor tissue samples were also collected during surgery and immediately snap-frozen in liquid nitrogen for RNA extraction. Total RNA was isolated using TRIzol reagent (Beyotime Biotechnology, Shanghai, China, China), and RNA concentration and purity were assessed by spectrophotometry and agarose gel electrophoresis.

The clinicopathological features of the selected CRC patients and healthy controls are summarized in **Table 1**.

**Table 1:** Clinicopathological features of metastatic CRC patients and healthy controls.

Clinicopathological features	CRC with metastasis (n = 10)	Healthy controls (n = 4)
Age at Sampling		
< 60 years	6	4
≥ 60 years	4	0
Tumor Location		–
Colon	7	–
Rectum	3	–
Histological Type		–
Moderately differentiated	8	–
Poorly/Neuroendocrine	2	–
Distant Metastasis		–
Peritoneal metastasis	6	–
Liver metastasis	3	–
Distant lymph node metastasis	1	–

All CRC cases included in this study had confirmed metastatic lesions. Peritoneal metastasis was identified by imaging and/or intraoperative observation. All patients were treatment-naïve at the time of sample collection. Healthy controls had no history of malignancy or systemic disease.

### 2.2 Cell culture

The human colorectal cancer cell line HCT116 was obtained from Wuhan Pricella Biotechnology Co., Ltd. (Wuhan, China). Cells were cultured in McCoy’s 5A medium (Gibco, USA) supplemented with 10% fetal bovine serum (FBS) (Nanjing SenBeiJia Biological Technology Co., Ltd., Nanjing, China), 1% penicillin (100 U/mL), and 1% streptomycin (100 µg/mL) (Beyotime Biotechnology, Shanghai, China Biotechnology Co., Ltd., Shanghai, China).

Human umbilical vein endothelial cells (HUVECs) were obtained from ScienCell Research Laboratories (Carlsbad, CA, USA; Cat. No. 8000) and cultured on gelatin-coated flasks in Endothelial Cell Medium (ECM, ScienCell, Cat. No. 1001) supplemented with 5% FBS, 1% endothelial cell growth supplement (ECGS), 100 U/mL penicillin, and 100 μg/mL streptomycin, according to the manufacturer’s instructions. Only passages 3–8 were used in all experiments to maintain endothelial characteristics, consistent with the protocol described in our previous work (22). Cell culture and maintenance procedures followed standard protocols for tumor and endothelial co-culture studies, as previously reported (22).

### 2.3 Cell viability assay

To evaluate the effect of tumor-derived conditioned medium (CM) on endothelial cell viability, HUVECs were seeded into 96-well plates at a density of 3 × 10³ cells/well in quintuplicates. After overnight adherence, cells were treated with CM collected from HCT116 cells transfected with miR-19b-3p mimic, inhibitor, or negative control, as previously described in our previous work (22), with minor modifications.

*Conditioned medium (CM) preparation:* HCT116 cells were transfected with either miR-19b-3p mimic, inhibitor, or miR-NC using Lipofectamine 3000 (Thermo Fisher Scientific) for 24 hours. Following transfection, cells were washed and cultured in serum-free McCoy’s 5A medium for an additional 48 hours. The supernatant was collected, centrifuged at 377 ×g for 5 minutes to remove cellular debris, and filtered through a 0.22 μm membrane filter. The resulting CM was used either undiluted (100%) or mixed with fresh ECM medium at a 1:1 ratio (50% CM). This CM preparation protocol was also applied to other experiments involving CM treatment, including migration, invasion, and Western blot assays, consistent with the methods in our previous work.

*Cell viability assay:* HUVECs were exposed to either 100% CM or different indicated concentrations of CM for 48 hours at 37 °C in a humidified incubator with 5% CO_2_. After treatment, 10 μL of Cell Counting Kit-8 (CCK-8; Labgic Technology Co., Ltd., Beijing, China) reagent was added to each well and incubated for an additional 2 hours. Absorbance was measured at 450 nm using a microplate reader (BioTek, USA).

### 2.4 Wound healing assay

A wound healing assay was performed to assess the migratory capacity of HCT116 and HUVECs, following a protocol established in our previous work (22) with minor modifications. Briefly, cells were seeded into 6-well plates at a density of 1 × 10^5^ cells/well and cultured until reaching approximately 90% confluence. A linear scratch was made in the center of each well using a sterile 200-μL pipette tip. Wells were gently washed with phosphate-buffered saline (PBS) to remove detached cells, and the culture medium was replaced with serum-free medium (without FBS or conditioned medium) to eliminate the influence of proliferation.

Immediately after scratching (0 h), images of the wound area were captured using a phase-contrast microscope. Cells were then incubated under standard conditions (37 °C, 5% CO_2_), and wound closure was monitored at multiple time points according to the migration kinetics of each cell type. For HCT116 cells, images were taken at 3, 6, and 12 hours post-scratch. For HUVECs, images were taken at 6, 12, and 24 hours post-scratch. These time points were selected based on preliminary experiments to reflect the distinct migration dynamics of each cell type. Wound closure was quantified using ImageJ software (NIH, USA). For each well, three random fields were selected, and in each field, five perpendicular lines were drawn across the wound area to measure the distance migrated. The migration rate (%) was calculated using the following formula:

Migration rate (%) = [(Initial wound width − Final wound width)/Initial wound width] × 100%

### 2.5 Transwell migration assay

To assess the migration ability of HUVECs in response to tumor-derived factors, a Transwell assay was performed using 24-well plates with 8 μm pore size polycarbonate inserts (Labgic Technology Co., Ltd., Beijing, China). A total of 600 μL of conditioned medium (CM) prepared from HCT116 cells (as described in Section 2.3) was added to the lower chamber. HUVECs (8 × 10³ cells/well) suspended in 100 μL of serum-free ECM medium were seeded in the upper chamber. The plates were incubated at 37 °C in 5% CO_2_ for 12 hours. Following incubation, non-migrated cells were gently removed from the upper surface of the membrane using a cotton swab. Migrated cells on the lower surface were fixed with 4% paraformaldehyde for 20 minutes, stained with 0.1% crystal violet for 10 minutes, and rinsed three times with PBS. Migrated cells were imaged under an optical microscope (Leica Microsystems, Germany). Quantification was performed using ImageJ software (NIH, USA) by analyzing three randomly selected fields per insert.

### 2.6 Cell transfection

To modulate miR-19b-3p expression, synthetic miRNA mimics and corresponding negative control mimics were obtained from Sangon Biotech (Shanghai, China). Transfections were performed using EL Transfection Reagent (TransGene Biotech, Beijing, China) according to the manufacturer’s protocol. The final concentration of miRNA mimics was 5 nM. Unless otherwise specified, cells were transfected for 24 hours prior to downstream applications, including conditioned medium collection (Section 2.3), migration assays (Section 2.5), and Western blot analysis (Section 2.7). The sequence of the miR-19b-3p mimic was: 5′-UGUGCAAAUCCAUGCAAAACUGA-3′. The negative control (NC) mimic sequences were as follows: sense strand, 5′-UCACAACCUCCUAGAAAGAGUAGA-3′; antisense strand, 5′-UCUACUCUUUCUAGGAGGUUGUGA-3′.

### 2.7 RNA extraction and quantitative real-time PCR

Total RNA was extracted from blood samples, HCT116 cells, and HUVECs using the RNAeasy™ Animal Total RNA Isolation Kit with Spin Column (Beyotime, Biotechnology Co., Ltd., Shanghai, China) following the manufacturer’s instructions. RNA concentration and purity were assessed using a NanoDrop spectrophotometer. Reverse transcription was conducted using the BeyoRT™ II First Strand cDNA Synthesis Kit (Beyotime Biotechnology, Shanghai, China Biotechnology). The reaction conditions were as follows: 25 °C for 10 min, 42 °C for 60 min, and 80 °C for 10 min. Quantitative real-time PCR (qPCR) was performed using SYBR Green Master Mix (Takara Bio, Japan) on a CFX96 Real-Time PCR Detection System (Bio-Rad, USA). Primer sequences are listed in **Table 2**. For mRNA quantification, GAPDH was used as the endogenous control, and relative gene expression was calculated using the 2^–ΔΔCt^ method. For miRNA quantification, miRNA-specific primers for miR-19b-3p and U6 were purchased as custom-designed assays from Sangon Biotech (Shanghai, China). The primer sequences were optimized by the manufacturer and are not publicly disclosed. U6 snRNA was used as the endogenous control for normalization.

**Table 2:** Primer sequences used for qPCR.

Gene	Primer	Sequence (5′–3′)
CPEB4	Forward	TGGGGATCAGCCTCTTCATA
	Reverse	CAATCCGCCTACAAACACCT
ZDHHC18	Forward	ACCGGCCTCTTCTTCGTCT
	Reverse	AACTGCCTGTGTTGTCGATCT
ZMYND11	Forward	GTGAAGCGCAGTATGGGTTG
	Reverse	TCGTCCTTTCTTTGCTCTTGG
MDM4	Forward	CAGCAGGTGCGCAAGGTGAA
	Reverse	CTGTGCGAGAGCGAGAGTCTG
GAPDH	Forward	GAAGGTGAAGGTCGGAGT
	Reverse	GAAGATGGTGATGGGATTTC
U6	Forward	CTCGCTTCGGCAGCACA
	Reverse	AACGCTTCACGAATTTGCGT

### 2.8 Western blotting

Western blotting was performed as previously described in our published study (22), with minor modifications. Briefly, HCT116 cells were lysed using RIPA buffer supplemented with protease and phosphatase inhibitors (Beyotime Biotechnology, Shanghai, China, Shanghai, China). Protein concentration was determined using the BCA Protein Assay Kit (Beyotime Biotechnology, Shanghai, China). Equal amounts of protein (10 μg per lane) were separated by 10% SDS-PAGE and transferred to PVDF membranes (Bio-Rad, USA). Membranes were blocked with 5% non-fat milk in TBST for 1 hour at room temperature and incubated overnight at 4 °C with the following primary antibodies: E-cadherin (#20874-1-AP, 1:1,000), α-SMA (#14395-1-AP, 1:1,000), and GAPDH (#60004-1-Ig, 1:5,000) (all from Proteintech, Wuhan, China). After washing, membranes were incubated with HRP-conjugated secondary antibodies (Beyotime Biotechnology, Shanghai, China) for 1 hour at room temperature. Protein bands were visualized using enhanced chemiluminescence (BeyoECL Plus, Beyotime Biotechnology, Shanghai, China) and quantified using ImageJ software. GAPDH served as the internal loading control.

### 2.9 RNA sequencing and bioinformatic analysis

HCT116 cells were transfected with either miR-19b-3p mimics or negative control mimics (NC). Six hours post-transfection, the culture medium was replaced, and cells were incubated for an additional 48 hours. Total RNA was extracted using TRIzol reagent (Beyotime Biotechnology, Shanghai, China) following the manufacturer’s instructions. RNA integrity and quality were assessed using an Agilent Bioanalyzer 2100 (Agilent Technologies, USA), and samples were stored in liquid nitrogen prior to library preparation. Three biological replicates per group were submitted for sequencing using the Illumina HiSeq™ 2500 platform.

Raw sequencing reads were aligned to the human reference genome (GRCh38) using HISAT2, and transcript abundance was quantified as TPM (transcripts per million) using StringTie. Differential gene expression was calculated using DESeq2, and genes with |fold change (FC)| > 1.2 and adjusted p-value (Benjamini–Hochberg method) < 0.05 were considered significantly differentially expressed. Gene Ontology (GO) and KEGG pathway enrichment analyses were performed using the clusterProfiler R package.

### 2.10 Clinical database analysis

Expression data for hsa-miR-19b-3p and relevant mRNAs were retrieved from The Cancer Genome Atlas (TCGA) colon adenocarcinoma (COAD) and rectum adenocarcinoma (READ) cohorts via the Broad GDAC Firehose portal (https://gdac.broadinstitute.org). A total of 631 samples in the COADREAD cohort were included. For mRNA analysis, FPKM values from RNA-seq data were used, while miRNA expression was extracted in counts per million (CPM) from the miRSeq dataset. Clinical parameters including tumor stage, metastasis status, and overall survival were also obtained from the Firehose database. Correlation analysis was performed using Spearman’s rank correlation, and survival analysis was conducted using the Kaplan–Meier method with log-rank test in R.

### 2.11 Public database and gene set enrichment analysis

Publicly available transcriptomic data were obtained from the Gene Expression Omnibus (GEO) database (https://www.ncbi.nlm.nih.gov/geo), specifically from dataset GSE155290, which contains transcriptomic profiles of human umbilical vein endothelial cells (HUVECs) transduced with either a SOX9 overexpression plasmid or an empty vector control, in order to study SOX9-associated gene expression programs in endothelial cells. Raw signal intensities were normalized and log_2_-transformed using the GCBI platform (https://www.expressanalyst.ca/). For differential expression analysis, the DESeq2 algorithm was applied with thresholds of |log_2_ fold change| > 0.26 (equivalent to fold change > 1.2) and adjusted p-value < 0.05. Gene Set Enrichment Analysis (GSEA) was performed to identify significantly enriched biological processes and cellular components. The analysis was conducted using the GSEA software (Broad Institute), with 1,000 gene set permutations. Enrichment scores were calculated based on the ranked list of differentially expressed genes, and significance was evaluated using normalized enrichment scores (NES), nominal p-values, and false discovery rate (FDR q-values). For SOX9-related analysis, transcriptomic data from SOX9-overexpressed HUVECs (GSE155290) were specifically analyzed to assess the enrichment of vesicle-associated gene sets, including “extracellular exosome” (Gene Ontology term).

### 2.12 Animal experiments

Male BALB/c mice (6–8 weeks old) were purchased from the Experimental Animal Center of Jiangsu University and housed under specific pathogen-free (SPF) conditions. All animal procedures were approved by the Institutional Animal Care and Use Committee of Jiangsu University (Approval No. JSDX-IACUC-AP-2023030513). Mice were randomly divided into three groups (n = 6 per group): (1) untransfected HCT116 (blank control), (2) HCT116 transfected with miR-19b-3p mimics, (3) HCT116 transfected with negative control miRNA (miR-NC). Each mouse received an intraperitoneal injection of 1 × 10^7^ cells suspended in 200 μL PBS. Body weight was monitored every 2 days for 14 days. At endpoint, mice were euthanized by CO_2_ inhalation (30% chamber volume per minute). The following organs were collected and analyzed: liver, lung, spleen, kidney, and peritoneum.

The following organs were collected and analyzed: heart, liver, spleen, lung, kidney, and gastrointestinal tract (including small and large intestines). Particular attention was paid to the serosal surface of the intestines, where visible metastatic nodules were most frequently observed. Tissues with visible lesions, especially from the intestinal surface, were fixed in 4% paraformaldehyde for 24 hours, embedded in paraffin, sectioned at 4–5 μm, and stained with H&E. Quantification of metastasis was performed by counting nodules in three random fields per section under a light microscope and calculating the metastatic index using Image-Pro Plus v8.0.

### 2.13 Histological and immunohistochemical analysis

Tissue sections were deparaffinized and rehydrated through a graded ethanol series. Antigen retrieval was performed by boiling the slides in 10 mM citrate buffer (pH 6.0) at 95 °C for 10 minutes. After natural cooling to room temperature, sections were blocked with 5% normal goat serum for 1 hour to reduce nonspecific binding. Slides were then incubated overnight at 4 °C with the following primary antibodies: E-cadherin (1:800, #20874-1-AP, Proteintech, Wuhan, China) and N-cadherin (1:800, #22018-1-AP, Proteintech, Wuhan, China).

After washing with PBS, sections were incubated with HRP-conjugated secondary antibodies (1:2000, #SA00001-2, Proteintech, Wuhan, China) for 1 hour at room temperature. Immunoreactive signals were developed using a DAB substrate kit and counterstained with hematoxylin. The stained sections were observed under a light microscope (Nikon or equivalent), and representative fields were selected for image capture at 200× magnification.

Quantification of protein expression was performed using the H-score method, which integrates both the staining intensity and the percentage of positive cells. For each tissue sample, three representative high-power fields (HPFs) were analyzed using Image-Pro Plus v8.0 (Media Cybernetics, USA). The H-score was calculated as previously described (24), using the following formula:

H-score = (% of weakly stained cells×1) + (% of moderately stained cells×2) + (% of strongly stained cells×3)

This yields a total score ranging from 0 to 300. Quantification was performed independently by two blinded investigators to ensure consistency. The mean H-score from three replicates was used for statistical analysis.

### 2.14 Statistical analysis

All experiments were independently repeated at least three times, unless otherwise stated. For each cell-based assay, technical replicates were performed in triplicate wells. Data are presented as mean ± standard deviation (SD). Prior to statistical testing, data normality was assessed using the Shapiro–Wilk test. For comparisons between two groups, Student’s t-test was used for normally distributed data, and the Mann–Whitney U test was used for non-parametric data. For comparisons among multiple groups, one-way ANOVA followed by Tukey’s *post hoc* test was applied when data were normally distributed, while Kruskal–Wallis test followed by Dunn’s multiple comparisons test was used for non-parametric data. Survival analysis was performed using the Kaplan–Meier method, and differences between curves were evaluated using the log-rank test. All statistical analyses were performed using GraphPad Prism version 9.0 (GraphPad Software, USA) and R version 4.3.1. A p-value < 0.05 was considered statistically significant.

In addition, protein–protein interaction (PPI) networks were constructed using Cytoscape v3.10, and hub genes were identified via the CytoHubba plugin.

## 3 Results

### 3.1 MiR-19b-3p is upregulated in colorectal cancer and associated with poor prognosis

**Figure 1:**
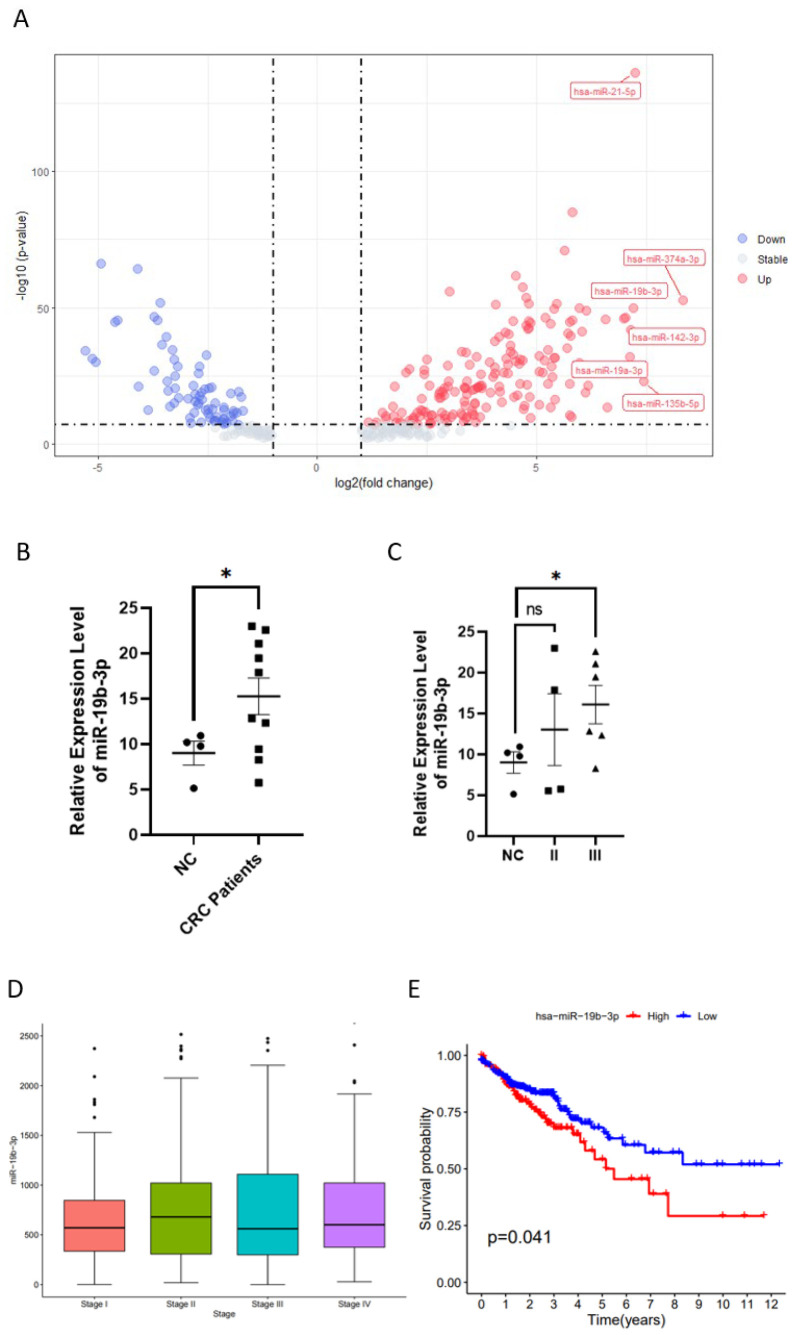
MiR-19b-3p is upregulated in colorectal cancer (CRC) and associated with poor prognosis. **(A)** Volcano plot showing differentially expressed miRNAs between CRC tumor and normal tissues from the TCGA-COAD and TCGA-READ datasets. Red and blue dots represent significantly upregulated and downregulated miRNAs, respectively (|log_2_ fold change| > 1, p < 0.05). **(B)** Relative expression of miR-19b-3p in peripheral blood from CRC patients (n = 10) and healthy volunteers (n = 4), measured by qRT-PCR. *p < 0.05 (Student’s t-test). **(C)** Circulating miR-19b-3p levels in healthy controls (n = 4), stage II CRC patients (n = 4), and stage III CRC patients (n = 6). *p < 0.05; ns, not significant (one-way ANOVA with Tukey’s test). **(D)** Boxplot showing miR-19b-3p expression across CRC stages I–IV in tumor tissues from TCGA. Data were extracted from miRSeq profiles and normalized to counts per million (CPM). Statistical analysis: Kruskal–Wallis test. **(E)** Kaplan–Meier survival analysis of overall survival in CRC patients (n = 631) from TCGA. Patients were stratified into high and low miR-19b-3p expression groups based on the median value. p = 0.041 (log-rank test).

We first analyzed miRNA expression profiles from the TCGA-COAD and TCGA-READ cohorts to identify dysregulated miRNAs in colorectal cancer (CRC). Among them, miR-19b-3p was one of the most significantly upregulated miRNAs in CRC tissues compared to normal tissues (**Figure 1A**). To validate these findings, we examined circulating miR-19b-3p levels in peripheral blood samples collected from CRC patients (n = 10) and healthy volunteers (n = 4). Quantitative real-time PCR (qRT-PCR) results revealed that miR-19b-3p expression was significantly elevated in CRC patients compared to controls (p < 0.05, **Figure 1B**). We further analyzed whether miR-19b-3p expression varied with disease stage. Although no formal correlation analysis was performed, miR-19b-3p levels appeared higher in stage II and III patients compared to healthy controls (**Figure 1C**). However, no statistically significant difference was observed between stage II and III patients. Consistent with these findings, analysis of TCGA-COAD and READ data showed that miR-19b-3p expression was elevated in CRC tumor tissues across all clinical stages (I–IV) relative to normal samples (**Figure 1D**). To explore the prognostic value of miR-19b-3p in CRC, we conducted Kaplan–Meier survival analysis based on TCGA patient data (n = 631). Patients were stratified into high and low expression groups using the median expression value of miR-19b-3p. The results showed that high miR-19b-3p expression was significantly associated with reduced overall survival (p = 0.041, **Figure 1E**). These results suggest that miR-19b-3p is upregulated in CRC tissues and peripheral blood and may be associated with advanced disease and poorer prognosis.

### 3.2 MiR-19b-3p enhances proliferation and migration of HCT116 cells and is associated with EMT

**Figure 2:**
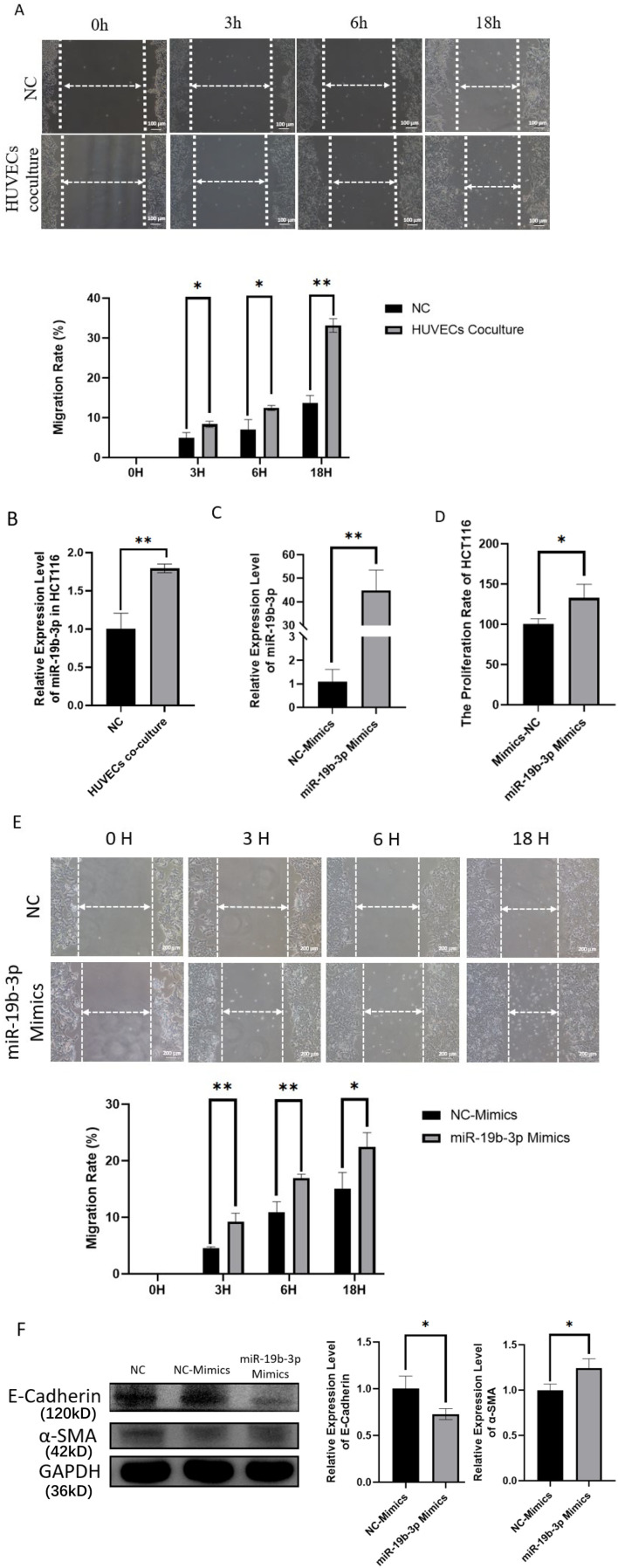
MiR-19b-3p enhances proliferation and migration of HCT116 cells and is associated with EMT. **(A)** Wound healing assay (scratch assay) showing increased migration of HCT116 cells after co-culture with HUVECs. **(B)** qRT-PCR analysis of miR-19b-3p expression in HCT116 cells co-cultured with HUVECs. **(C)** Verification of miR-19b-3p overexpression in HCT116 cells transfected with miR-19b-3p mimics by qRT-PCR. **(D)** CCK-8 assay showing enhanced proliferation of HCT116 cells overexpressing miR-19b-3p. **(E)** Wound healing assay showing increased migration of HCT116 cells following miR-19b-3p overexpression. **(F)** Western blot analysis of EMT markers in miR-19b-3p-overexpressing HCT116 cells. E-cadherin (120 kD) was downregulated, while α-SMA (42 kD) was upregulated. Relative band intensity was quantified by grayscale analysis. Data are presented as mean ± SD from three independent experiments (*p < 0.05; ** *p* < 0.01).

To investigate the potential role of miR-19b-3p in colorectal cancer cell migration, we first utilized a co-culture system of HCT116 cells and HUVECs. Wound healing assays (scratch assay) revealed that HCT116 cells co-cultured with HUVECs exhibited significantly enhanced migration ability compared to monocultured controls (**Figure 2A**). Notably, miR-19b-3p expression in HCT116 cells was upregulated under co-culture conditions, as determined by qRT-PCR (**Figure 2B**), suggesting that interaction with endothelial cells may induce miR-19b-3p expression. To assess the direct functional effects of miR-19b-3p on CRC cells, we transfected HCT116 cells with synthetic miR-19b-3p mimics. Successful overexpression was confirmed by qRT-PCR (**Figure 2C**). Functional assays demonstrated that miR-19b-3p overexpression significantly promoted cell proliferation (**Figure 2D**) and enhanced migration in scratch assays (**Figure 2E**), compared to the negative control group. To explore the underlying mechanisms, we examined markers of epithelial–mesenchymal transition (EMT). Western blot analysis showed that E-cadherin (120 kD), an epithelial marker, was downregulated, while α-SMA (42 kD), a mesenchymal marker, was upregulated in miR-19b-3p-overexpressing cells (**Figure 2F**). These results suggest that miR-19b-3p may enhance the invasive phenotype of CRC cells by promoting EMT-like changes.

### 3.3 Transcriptomic analysis suggests that miR-19b-3p may promote EMT via the MAPK signaling pathway

**Figure 3:**
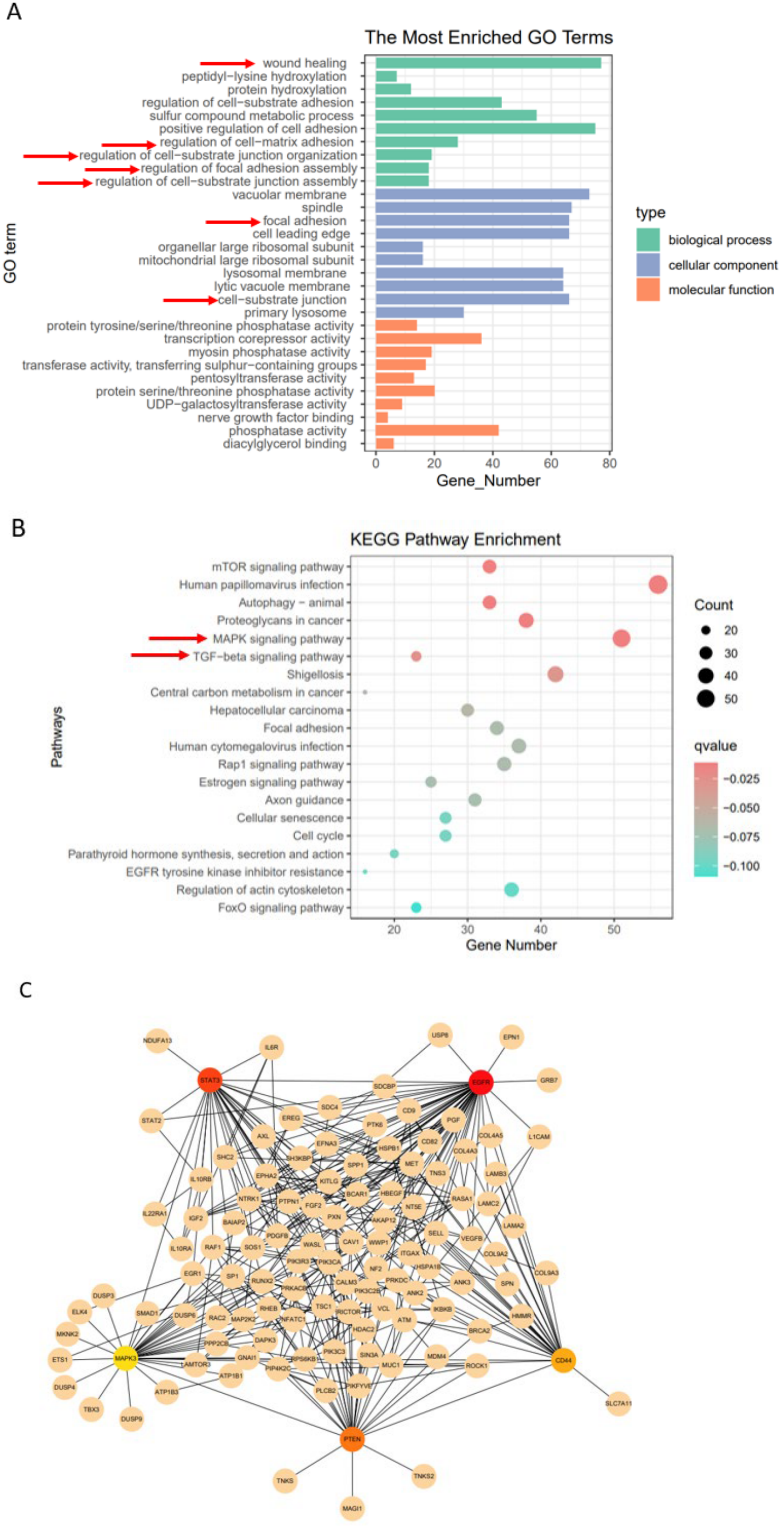
Transcriptomic profiling suggests involvement of the MAPK signaling pathway in miR-19b-3p-induced EMT. **(A)** Gene Ontology (GO) enrichment analysis of differentially expressed genes (DEGs) between miR-19b-3p-overexpressing and control HCT116 cells. DEGs were defined as |log_2_(fold change)| > 1 and FDR < 0.05. **(B)** KEGG pathway enrichment analysis of the same DEG set, highlighting significant enrichment in MAPK and TGF-β signaling pathways. **(C)** Protein–protein interaction (PPI) network visualization of DEGs constructed using the STRING database and analyzed with the cytoHubba plugin in Cytoscape. The top five hub genes (EGFR, STAT3, PTEN, CD44, MAPK3) are shown. Red nodes indicate the gene with the highest centrality score; yellow denotes the lowest.

To further explore the molecular mechanisms by which miR-19b-3p promotes EMT in colorectal cancer cells, we performed RNA sequencing on HCT116 cells transfected with miR-19b-3p mimics or negative control (NC). Differentially expressed genes (DEGs) were identified using the threshold of |log_2_(fold change)| > 1 and adjusted p-value (FDR) < 0.05. A total of 812 genes were significantly upregulated, and 673 were downregulated in the miR-19b-3p-overexpressing group compared to the control. Gene Ontology (GO) enrichment analysis revealed that DEGs were mainly involved in cell migration, extracellular matrix organization, and signal transduction (**Figure 3A**). Kyoto Encyclopedia of Genes and Genomes (KEGG) pathway analysis further showed significant enrichment in the MAPK signaling pathway and TGF-β signaling pathway (**Figure 3B**), both of which are known to regulate EMT. Given that MAPK signaling has been previously implicated in EMT regulation [26], we constructed a protein–protein interaction (PPI) network using the STRING database and visualized the network in Cytoscape. Using the cytoHubba plugin, we identified the top five hub genes based on degree centrality: EGFR, STAT3, PTEN, CD44, and MAPK3 (**Figure 3C**). These genes are functionally involved in MAPK and EMT-related signaling. Although these transcriptomic and bioinformatic results suggest that miR-19b-3p may regulate EMT through the MAPK pathway, further experimental validation is required to confirm these findings.

### 3.4 ZMYND11 is a putative downstream target of miR-19b-3p in HCT116 cells

**Figure 4:**
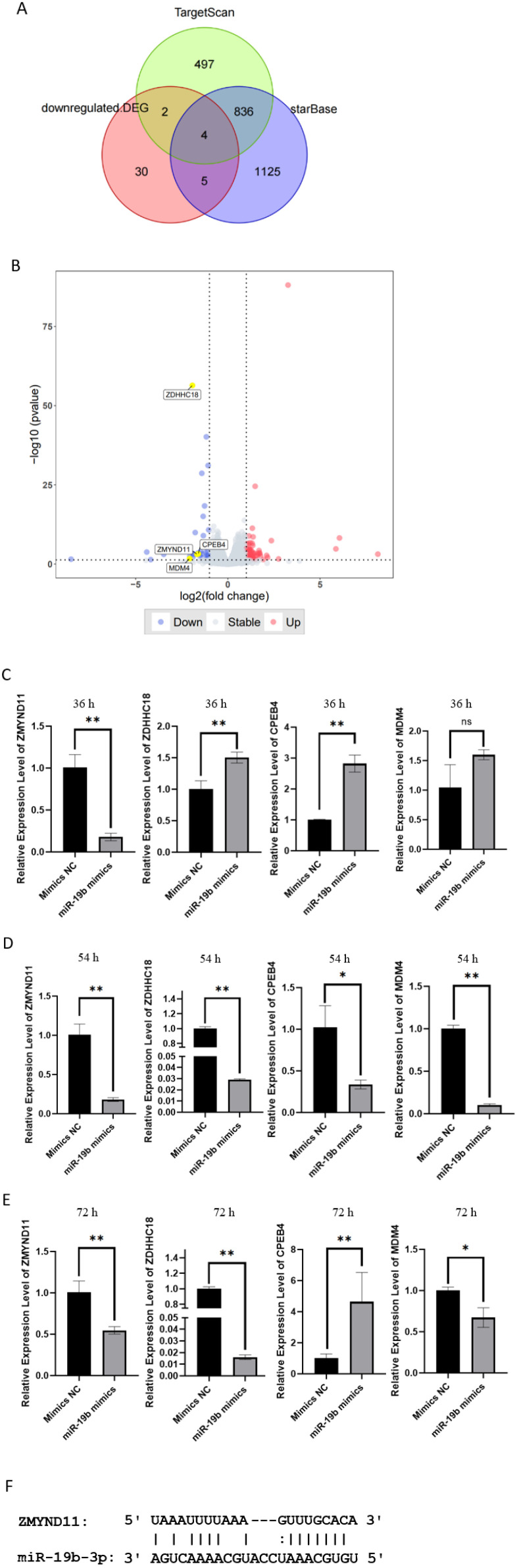
Identification of ZMYND11 as a putative target of miR-19b-3p in HCT116 cells. **(A)** Venn diagram showing overlap of predicted miR-19b-3p targets from TargetScan and starBase with genes downregulated in RNA-seq (|fold change| > 2, p < 0.05). **(B)** Volcano plot of differentially expressed genes in miR-19b-3p-overexpressing versus control HCT116 cells, highlighting candidate targets. **(C–E)** qRT-PCR analysis of mRNA expression levels of the four candidate genes (ZMYND11, ZDHHC18, CPEB4, and MDM4) at 36 h **(C)**, 54 h **(D)**, and 72 h **(E)** after transfection with miR-19b-3p mimics. **(F)** Predicted binding site of miR-19b-3p in the 3′ untranslated region (3′-UTR) of ZMYND11. Data are presented as mean ± SD from three independent experiments. *p < 0.05; **p < 0.01.

To identify potential downstream targets of miR-19b-3p, we performed integrative analysis using two microRNA target prediction tools: TargetScan (25) and starBase (26). To narrow down potential functional targets, we cross-referenced predicted genes with those downregulated more than 2-fold (|fold change| > 2, *p* < 0.05) in our RNA-seq dataset of miR-19b-3p-overexpressing HCT116 cells. This yielded four candidate targets: ZMYND11, ZDHHC18, CPEB4, and MDM4 (**Figures 4A, B**).

Among these, ZMYND11 has been previously reported as a tumor suppressor in various cancer types, including breast and prostate cancer (27, 28). However, its functional role and targeting by miR-19b-3p in colorectal cancer require further validation.

To evaluate whether miR-19b-3p modulates the expression of these candidates, we performed qRT-PCR analysis at 36, 54, and 72 hours after transfection of HCT116 cells with miR-19b-3p mimics. Although some variability was observed for ZDHHC18, CPEB4, and MDM4, only ZMYND11 displayed consistent and statistically significant downregulation across all time points (**Figures 4C–E**), suggesting it may be a biologically relevant target of miR-19b-3p.

Furthermore, silico prediction revealed a putative miR-19b-3p binding site within the 3′ untranslated region (3′-UTR) of ZMYND11 mRNA (**Figure 4F**). However, it is important to note that direct interaction between miR-19b-3p and ZMYND11 has not been experimentally validated. Future studies incorporating luciferase reporter assays and mutant 3′-UTR co-transfection experiments are needed to confirm this regulatory relationship.

### 3.5 HCT116-conditioned medium enhances HUVEC proliferation and migration and increases miR-19b-3p expression

MiR-19b-3p has been reported as an endothelial cell-specific microRNA (21). In this study, we examined whether colorectal cancer cells (HCT116) influence the behavior of human umbilical vein endothelial cells (HUVECs) through conditioned medium and whether miR-19b-3p expression is involved in this process.

To evaluate this, HUVECs were seeded in 96-well plates (100 μL/well) and cultured overnight. The next day, the medium was replaced with 100 μL of HCT116-conditioned medium (CM) at varying concentrations (0%, 10%, 20%, 40%, 60%, 80%, and 100%), each diluted with endothelial cell medium (ECM) to a final volume of 100 μL per well. The 0% CM group (only ECM basal medium without serum) was defined as the negative control (NC), while a separate group of HUVECs cultured in complete ECM medium (200 μL) served as the positive control (PC). Five replicates were set per group. To minimize edge effects, 200 μL PBS was added to all peripheral wells. Cells were cultured for 48 hours at 37 °C in 5% CO_2_ before assays.

**Figure 5:**
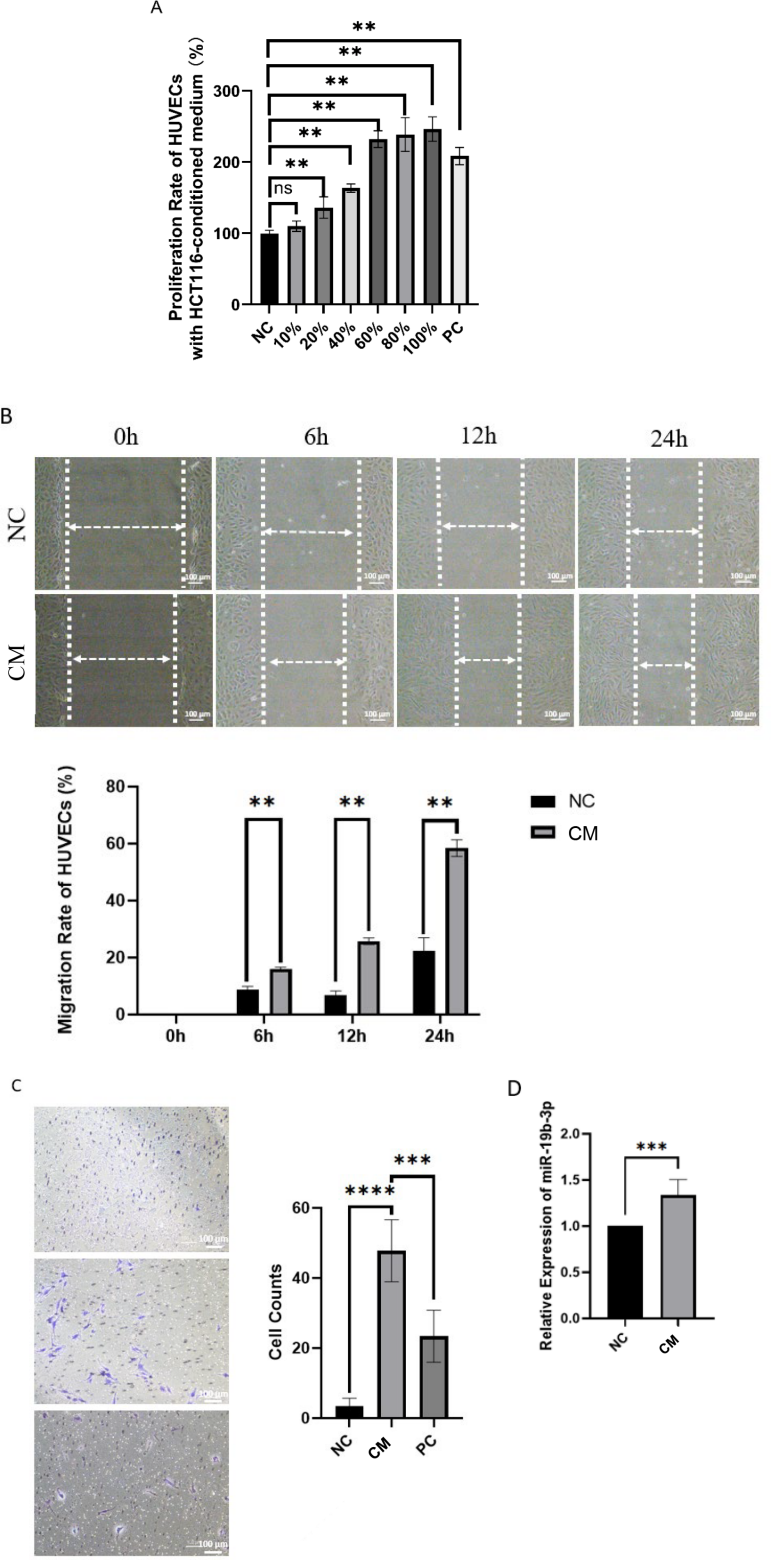
Effects of HCT116-conditioned medium on HUVEC proliferation, migration, and miR-19b-3p expression. **(A)** CCK-8 assay showing HUVEC viability after 48 h exposure to varying concentrations of HCT116-conditioned medium (CM). **(B)** Wound healing assay assessing HUVEC migration after treatment with HCT116-CM. **(C)** Transwell migration assay showing increased motility of HUVECs in response to HCT116-CM. **(D)** Relative expression of miR-19b-3p in HUVECs after 48 h exposure to HCT116-CM. Data are presented as mean ± SD from three independent experiments. *p* < 0.05; ** *p* < 0.01; ***p < 0.001 and ****p < 0.0001 vs. NC group.

Cell proliferation was assessed using the CCK-8 assay. Results showed a concentration-dependent increase in HUVEC viability with increasing proportions of HCT116-conditioned medium, compared to the NC group (**Figure 5A**).

To further assess the impact on cell motility, wound healing and Transwell migration assays were performed. HUVECs exposed to HCT116-conditioned medium showed significantly enhanced wound closure (**Figure 5B**) and increased transmembrane migration (**Figure 5C**), indicating that factors secreted by HCT116 cells promote endothelial cell migration.

Lastly, we analyzed miR-19b-3p expression in HUVECs after exposure to HCT116-conditioned medium. qRT-PCR results revealed a significant upregulation of miR-19b-3p in treated HUVECs compared to the NC group (**Figure 5D**), suggesting that HCT116-derived factors may influence endothelial cell function in part through modulation of miR-19b-3p expression.

However, it should be noted that while miR-19b-3p expression was elevated, the current data do not directly demonstrate that miR-19b-3p mediates the communication between HCT116 cells and HUVECs. Further mechanistic studies (e.g., miRNA inhibition or overexpression) are needed to confirm a causal role.

### 3.6 MiR-19b-3p promotes EndMT in HUVECs and is associated with vesicle-related gene expression

**Figure 6:**
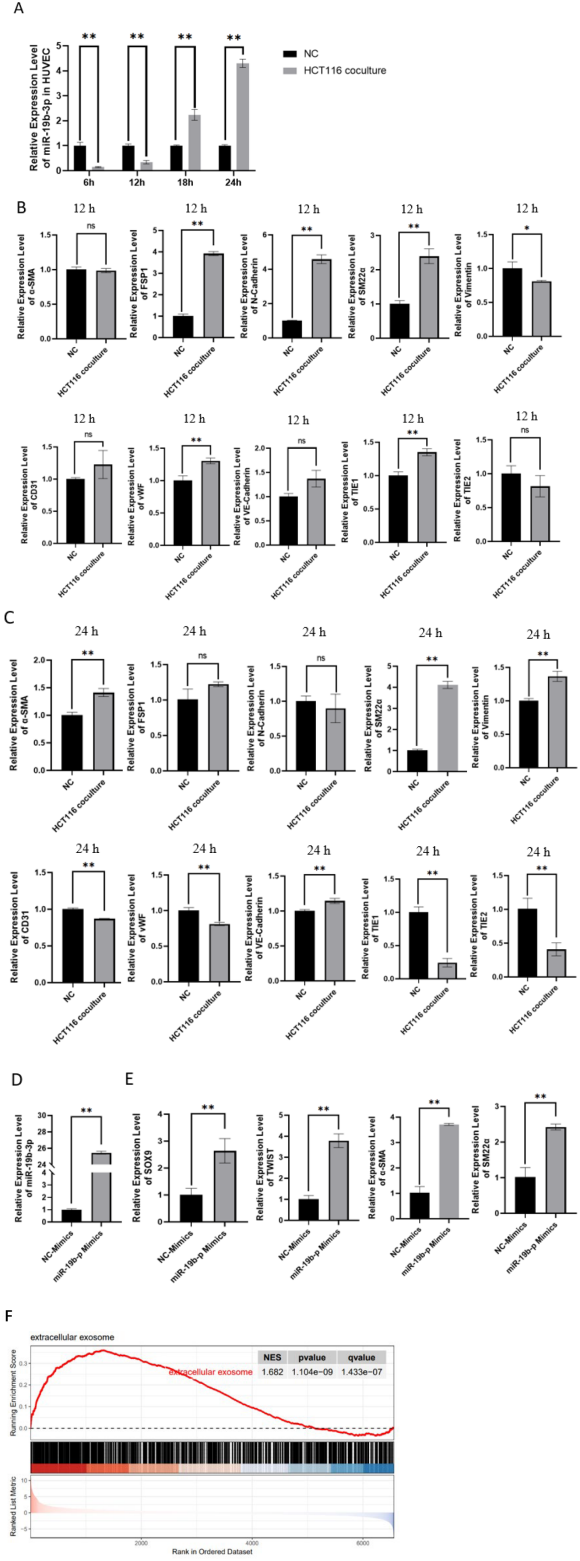
MiR-19b-3p promotes EndMT and is associated with exosome-related gene expression. **(A)** The expressions of miR-19b-3p in HUVECs co-cultured with HCT116 cells increased in a time-dependent manner, as determined by qRT-PCR at 6 h, 12 h, 18 h, and 24 h. **(B)** Expressions of endothelial-to-mesenchymal transition (EndMT) markers in HUVECs after 12 h of co-culture with HCT116 cells. Several mesenchymal markers (e.g., FSP1, N-cadherin, SM22α, Vimentin) were significantly upregulated, while most endothelial markers remained unchanged. **(C)** Expressions of EndMT-related genes in HUVECs after 24 h of co-culture with HCT116 cells. At this time point, mesenchymal markers showed further upregulation (e.g., α-SMA, SM22α), and endothelial markers (e.g., VE-cadherin, CD31, TIE1/2) were significantly downregulated, indicating progression of EndMT. **(D)** Transfection efficiency of miR-19b-3p mimics in HUVECs was confirmed by qRT-PCR. **(E)** Overexpression of miR-19b-3p in HUVECs induced significant upregulation of SOX9, TWIST1, α-SMA, and SM22α, supporting its role in promoting EndMT. **(F)** Gene Set Enrichment Analysis (GSEA) of transcriptomic data from SOX9-overexpressed HUVECs (GSE155290) revealed significant enrichment of the “extracellular exosome” gene set (NES = 1.682, p = 1.104×10^-9^, q = 1.433×10^-7^), suggesting a potential association between SOX9 signaling and vesicle-related gene expression programs. All data are presented as mean ± SD. * *p* < 0.05; ** *p* < 0.01.

In the HCT116-HUVEC co-culture system, the expression of miR-19b-3p in HUVECs was significantly upregulated in a time-dependent manner (**Figure 6A**). At 12 hours of co-culture, several mesenchymal markers such as FSP1, N-cadherin, SM22α, and Vimentin were significantly increased, while most endothelial markers remained unchanged (**Figure 6B**). By 24 hours, the expression of mesenchymal markers including α-SMA, SM22α, and Vimentin was further elevated, while endothelial markers such as VE-cadherin, CD31, and TIE1/2 were significantly downregulated (**Figure 6C**), indicating progressive EndMT.

To further validate the regulatory role of miR-19b-3p, HUVECs were transfected with miR-19b-3p mimic. Overexpression of miR-19b-3p markedly upregulated SOX9, TWIST1, α-SMA, and SM22α (**Figures 6D, E**), supporting its role in promoting EndMT.

Furthermore, GSEA analysis of transcriptomic data from SOX9-overexpressed HUVECs (GSE155290) showed significant enrichment of the extracellular exosome gene set (NES = 1.682, p = 1.104×10^-9^, q = 1.433×10^-7^) (**Figure 6F**). Although exosomes were not directly isolated in this study, these results suggest a potential association between SOX9-induced EndMT and vesicle-related gene expression, which warrants further validation.

### 3.7 MiR-19b-3p promoted CRC metastasis *in vivo* mice model

**Figure 7:**
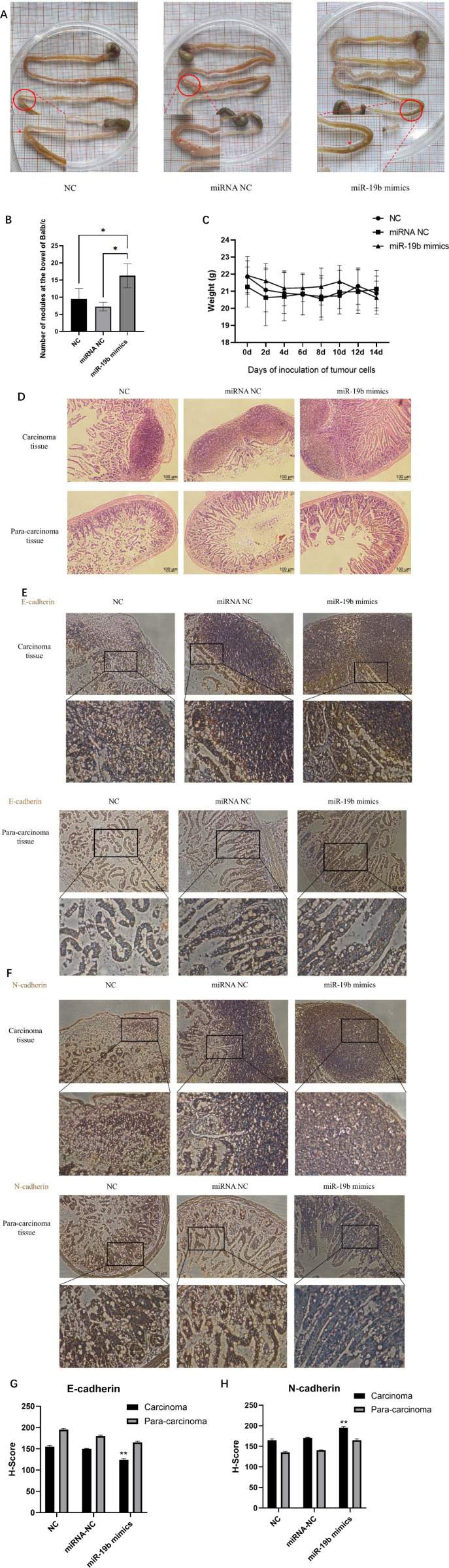
miR-19b-3p promotes colorectal cancer metastasis *in vivo* and induces epithelial–mesenchymal transition (EMT). **(A)** Representative gross images of the peritoneal cavity and intestinal tracts from BALB/c mice 14 days after intraperitoneal injection of untransfected HCT116 cells (NC group), HCT116 cells transfected with negative control miRNA (miRNA NC group), or HCT116 cells transfected with miR-19b-3p mimics (miR-19b mimics group). Multiple tumor nodules were observed along the serosal surface of the intestine in the miR-19b mimics group (highlighted by red circles), whereas fewer and smaller nodules were present in control groups. Enlarged insets show the demarcation between tumor lesions and surrounding normal tissue. **(B)** Quantification of visible tumor nodules. Mice in the miR-19b mimics group developed significantly more nodules than those in the NC and miRNA NC groups (*P* < 0.05). Data are presented as mean ± SD (n = 6 mice per group). **(C)** Body weight monitoring throughout the 14-day experimental period. All groups exhibited mild fluctuations with no significant differences. No ascites was observed in any group at necropsy. **(D)** Hematoxylin and eosin (H&E) staining of carcinoma and adjacent para-carcinoma tissues. Tumors from the miR-19b mimics group displayed irregular glandular architecture, nuclear pleomorphism, and deeper stromal invasion. Para-carcinoma tissues retained normal epithelial morphology. Scale bar = 100 μm. **(E)** Immunohistochemical staining of E-cadherin. Strong membranous E-cadherin expression was observed in para-carcinoma tissues across all groups. In carcinoma tissues, E-cadherin expression was markedly reduced in the miR-19b mimics group, indicating loss of epithelial characteristics. Scale bar = 50 μm. **(F)** Immunohistochemical staining of N-cadherin. Weak N-cadherin expression was observed in para-carcinoma tissues, whereas strong cytoplasmic and membranous N-cadherin expression was detected in carcinoma tissues of the miR-19b mimics group, indicating mesenchymal transition. Scale bar = 50 μm. **(G, H)** Semi-quantitative analysis of E-cadherin **(G)** and N-cadherin **(H)** expression using H-score quantification. In carcinoma tissues, the E-cadherin H-score was significantly lower and the N-cadherin H-score significantly higher in the miR-19b mimics group compared to the NC and miRNA NC groups (**P < 0.05). Para-carcinoma tissues consistently showed high E-cadherin and low N-cadherin expression. Data represent mean ± SD from three biological replicates.

To evaluate the effect of miR-19b-3p on colorectal cancer (CRC) metastasis *in vivo*, an intraperitoneal injection model was established using HCT116 cells. Mice were divided into three groups: those injected with untransfected HCT116 cells (NC group), HCT116 cells transfected with negative control miRNA (miRNA NC group), and HCT116 cells transfected with miR-19b-3p mimics (miR-19b mimics group). Anatomical examination revealed that mice in the miR-19b mimics group developed significantly more metastatic lesions on the surface of the gastrointestinal tract compared with the NC and miRNA NC groups (**Figures 7A, B**, *P* < 0.05). Throughout the experiment, body weight was monitored as an indicator of overall health status and tumor burden. All groups exhibited mild fluctuations in weight with no significant differences (**Figure 7C**). Importantly, no ascites was observed in any group at necropsy, indicating that the increased metastatic burden in the miR-19b mimics group was not associated with overt cachexia or fluid accumulation.

Histopathological analysis by H&E staining further confirmed these findings. Tumors from the miR-19b mimics group exhibited more aggressive features, including irregular glandular architecture, prominent nuclear pleomorphism, and deeper stromal invasion, whereas tumors in the control groups displayed compact and less invasive morphology. Para-carcinoma tissues showed preserved epithelial structure with no evidence of malignancy (**Figure 7D**).

To explore the mechanism underlying enhanced metastasis, immunohistochemical (IHC) staining was performed for key epithelial–mesenchymal transition (EMT) markers. E-cadherin, an epithelial marker, was strongly expressed at the cell membrane in para-carcinoma tissues across all groups. In contrast, its expression was markedly reduced in carcinoma tissues, particularly in the miR-19b mimics group compared to NC and miRNA NC groups (**Figure 7E**).

To provide quantitative support for the observed staining differences, semi-quantitative H-score analysis was conducted. In carcinoma tissues, the E-cadherin H-score was significantly lower in the miR-19b mimics group (mean 124 ± 2.5) compared to the NC group (155 ± 3.0) and miRNA NC group (150 ± 1.5) (*P* < 0.05). Conversely, in para-carcinoma tissues, E-cadherin expression remained relatively high across all groups (**Figure 7G**).

In contrast, N-cadherin expression was significantly upregulated in carcinoma tissues from the miR-19b mimics group, while remaining low in para-carcinoma tissues and in control groups (**Figure 7F**). H-score quantification revealed that N-cadherin levels in the carcinoma tissue of the miR-19b mimics group (mean 195 ± 3.0) were notably higher than those of the NC (165 ± 2.5) and miRNA NC groups (170 ± 2.0) (*P* < 0.05), further supporting the occurrence of EMT (**Figure 7H**).

In summary, these *in vivo* results demonstrate that miR-19b-3p overexpression promotes peritoneal metastasis of CRC cells, likely through the induction of EMT, as evidenced by increased tumor nodule formation, decreased E-cadherin, and increased N-cadherin expression, with supporting H-score quantification of IHC staining.

## 4 Discussion

miR-19b-3p, a member of the oncogenic miR-17~92 cluster, has been previously reported to promote proliferation in colorectal cancer (CRC) (18). However, its role in regulating tumor metastasis and microenvironmental interactions remained poorly understood. In this study, we provide both *in vitro* and *in vivo* evidence that miR-19b-3p facilitates CRC progression by promoting epithelial–mesenchymal transition (EMT) in tumor cells and endothelial–mesenchymal transition (EndMT) in vascular endothelial cells, highlighting its dual role in modulating tumor–stromal crosstalk.

Our *in vivo* metastasis model showed that overexpression of miR-19b-3p significantly increased peritoneal tumor burden without inducing ascites or body weight loss, suggesting enhanced metastatic dissemination without systemic toxicity. Histological and immunohistochemical analyses confirmed that miR-19b-3p promotes EMT, as evidenced by architectural disorganization, downregulation of E-cadherin, and upregulation of N-cadherin (20).

Mechanistically, our RNA-seq and pathway enrichment analyses revealed that miR-19b-3p may regulate EMT through both the TGF-β and MAPK signaling pathways (29–33). These pathways are well-established mediators of EMT in various cancers. For example, activation of the ERK-MAPK pathway has been linked to EMT induction via transcription factors such as ZEB1 in pancreatic and melanoma models (32, 34). Notably, natural compounds such as curcumin have been reported to regulate EMT through these pathways (35) and modulate miR-19b-3p expression (36), suggesting a potential link between therapeutic agents and miRNA-driven EMT regulation (37–41).

Among the predicted targets of miR-19b-3p, ZMYND11 emerged as the only gene consistently downregulated across datasets. ZMYND11 is a known tumor suppressor involved in chromatin regulation and MAPK pathway modulation (28, 42). Previous studies have shown that ZMYND11 inhibits EGFR signaling and suppresses migration and invasion in renal cancer. In our study, EGFR is the top hub gene in the protein–protein interaction (PPI) network. Additionally, MAPK3 is another ranked hub gene and belongs to the same subnetwork as ETS1, a key EMT transcription factor (43). These findings suggest that miR-19b-3p may promote EMT via the ZMYND11/EGFR/ETS1 axis. However, although ZMYND11 expression was reduced upon miR-19b-3p overexpression, a direct regulatory interaction remains to be validated. Future studies incorporating luciferase reporter assays and site-directed mutagenesis are warranted to confirm this potential miRNA–target relationship.

In addition to its cell-autonomous effects, miR-19b-3p appears to mediate dynamic crosstalk between CRC cells and endothelial cells. In our co-culture system, HCT116 cells induced EndMT in HUVECs, as evidenced by increased expression of α-SMA, SM22α, and transcription factors TWIST1 and SOX9 (12). SOX9 is known to transcriptionally regulate SNAI1/2, which in turn controls mesenchymal marker expression during EndMT. These results suggest that miR-19b-3p may promote EndMT via SOX9 activation. Furthermore, overexpression of miR-19b-3p in HUVECs significantly enhanced mesenchymal marker expression, indicating a functional role in endothelial plasticity.

Interestingly, we observed a reciprocal increase in miR-19b-3p levels in both HCT116 and HUVECs during co-culture, suggesting a potential positive feedback loop between EMT and EndMT. Although our results show that miR-19b-3p expression is upregulated in HUVECs upon exposure to HCT116-conditioned medium, causality has not been definitively established. Future studies using miR-19b-3p knockdown or overexpression in co-culture and transwell systems are needed to dissect its role in intercellular communication.

In line with previous findings that endothelial cells can promote tumor aggressiveness through Notch1 or ROBO1 signaling (9, 10), our findings suggest that endothelial cells may also contribute to CRC metastasis via miR-19b-3p-dependent mechanisms. miR-19b-3p has been detected in endothelial-derived exosomes (44), which are known to mediate tumor–stromal interactions and respond to inflammatory stimuli. This raises the possibility that exosomal miR-19b-3p may serve as a communication vehicle between endothelial and cancer cells, further amplifying metastatic signaling (45).

**Figure 8:**
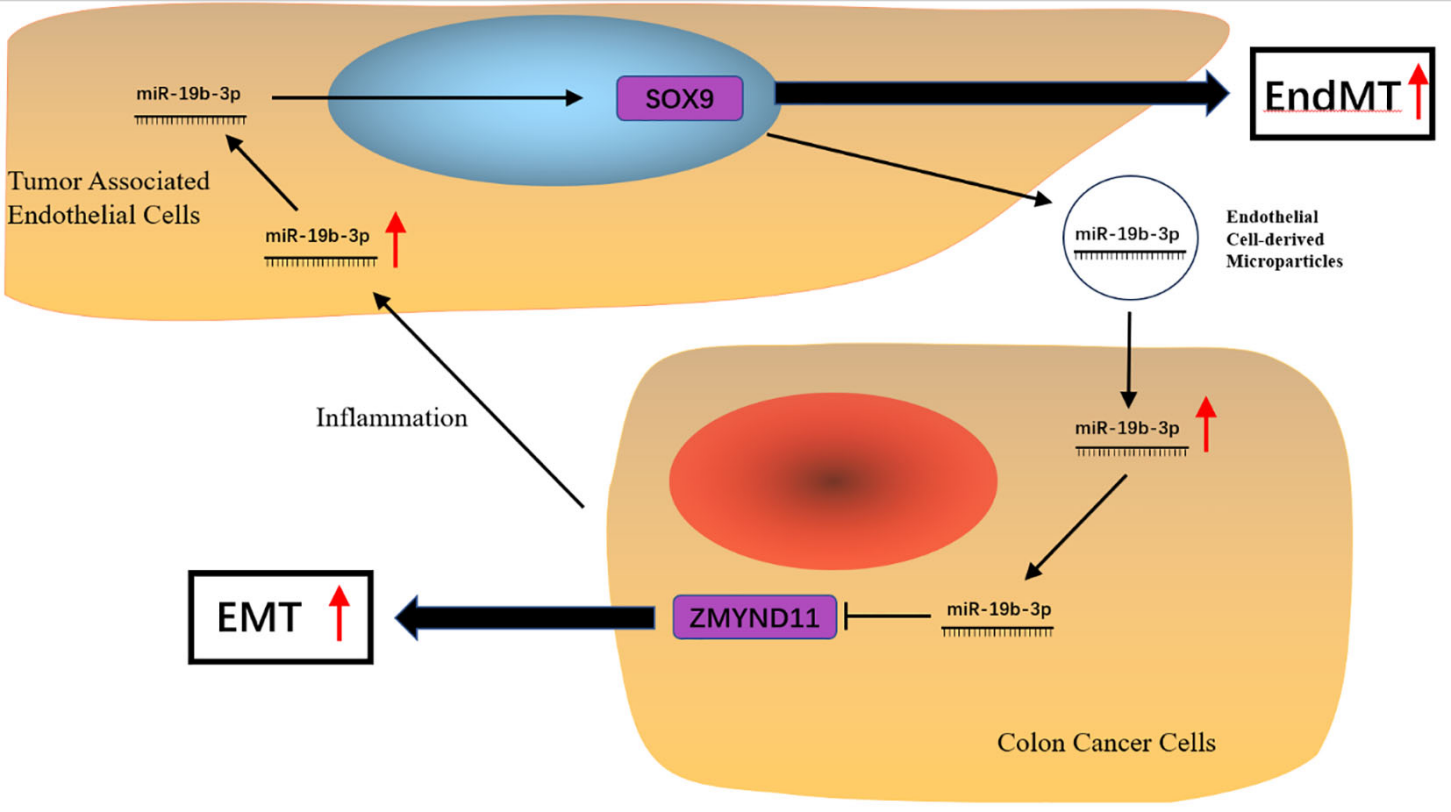
Proposed working model of miR-19b-3p–mediated EMT–EndMT crosstalk in colorectal cancer. Tumor-associated endothelial cells upregulate miR-19b-3p in HCT116 cancer cells, promoting EMT via ZMYND11 suppression and MAPK signaling activation. In turn, HCT116 cells enhance miR-19b-3p in endothelial cells, inducing SOX9-mediated EndMT and increasing exosome secretion. This bidirectional feedback loop may drive CRC metastasis.

These findings suggest that miR-19b-3p may serve as both a biomarker and a therapeutic target in colorectal cancer by mediating tumor–endothelial crosstalk and enhancing metastatic potential. A schematic model summarizing the proposed mechanism is shown in **Figure 8**, which illustrates the bidirectional interaction between cancer cells and endothelial cells mediated by miR-19b-3p, involving SOX9, ZMYND11, and exosomal signaling pathways.

However, several limitations of this study should be noted. Although ZMYND11 was identified as a potential downstream target of miR-19b-3p (42), the direct interaction remains to be confirmed, and future studies incorporating luciferase reporter assays and mutagenesis are required to validate this regulatory axis. In addition, while elevated miR-19b-3p levels were observed in HUVECs upon co-culture with HCT116 cells, causal involvement in endothelial–tumor communication was not fully established. Functional studies involving miR-19b-3p knockdown in co-culture systems would help clarify its mechanistic role. Moreover, the peritoneal injection model used in this study may not fully recapitulate the natural course of colorectal cancer metastasis, and orthotopic or spontaneous metastasis models are needed to further corroborate our findings. Lastly, the contribution of exosomal miR-19b-3p to intercellular transfer remains speculative at this stage and warrants further investigation (44, 45).

Despite these limitations, our data provide valuable insights into the role of miR-19b-3p in modulating colorectal cancer metastasis and tumor–endothelial interactions.

In conclusion, this study demonstrates that miR-19b-3p enhances CRC metastasis by promoting both EMT and EndMT, potentially through a self-reinforcing loop. Given its elevated levels in patient blood and its dual regulatory role, miR-19b-3p holds promise as a diagnostic biomarker and therapeutic target. Incorporating miR-19b-3p into future biomarker panels or liquid biopsy strategies may improve early detection and treatment of metastatic colorectal cancer.

## Data Availability

The original contributions presented in the study are included in the article/supplementary material. Further inquiries can be directed to the corresponding author.
